# Ge_2_Sb_2_Se_4_Te‐Based Optical Switch with Ultra‐High Contrast Ratio by Multilayer Fabry–Perot Cavity

**DOI:** 10.1002/advs.202412499

**Published:** 2025-02-14

**Authors:** Zhiyang Tang, Chensheng Li, Ruhao Pan, Bo Wang, Yunan Liu, Qianyu Wang, Junhao Tan, Yuan Xiang, Haifang Yang, Junjie Li

**Affiliations:** ^1^ Beijing National Laboratory for Condensed Matter Physics Institute of Physics Chinese Academy of Sciences Beijing 100190 China; ^2^ School of Physical Sciences University of Chinese Academy of Sciences Beijing 100049 China

**Keywords:** high contrast ratio, optical switch, phase change materials, scalable fabrication, tunable multilayer cavity

## Abstract

Optical switches always desire a high contrast ratio in optical logic circuits and optical communication devices. Although phase change materials are widely applied to design optical switches for their rapid phase change, the high contrast ratio is still hardly achieved for the intrinsic loss of traditional PCMs. Here, the study demonstrates an optical switch with an ultra‐high contrast ratio by introducing the Ge_2_Sb_2_Se_4_Te (GSST) into a multilayer film structure treated as a Fabry–Perot (FP) cavity. The reflectance of the multilayer system can be actively tuned by the phase change of GSST for the FP resonance to achieve an optical switch response. By designing the thicknesses of each layer in the multilayer film, the operating wavelength and the contrast ratio of the switch can be precisely controlled, and an ultra‐high contrast ratio of 2410/735 (simulation/experiment) for the optical switch is constructed in the near‐infrared band. The development of the GSST‐based optical switch paves a new way to realize a high optical contrast ratio and offers a new strategy for high‐performance optical devices.

## Graphical abstract

This work designs a multilayered optical switch with phase change material Ge_2_Sb_2_Se_4_Te based on the Fabry–Perot resonances changing under the phase change process. By reasonably selecting the thickness of the multilayer, an ultrahigh contrast ratio (735) of the optical switch is achieved in the near‐infrared band. The result provides fresh possibilities for the next‐generation active optical systems.

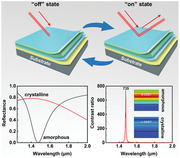

## 1 Introduction

Micro/nano‐optical devices, which can achieve light emission, modulation, absorption, and sensing under limited space, have flourished and been widely researched in recent years for their irreplaceable advantages of small footprint, high performance, and compact integration compared to conventional optical components.^[^ ^1^ ^]^ It is generally believed that micro/nano‐optical devices can be utilized in all kinds of next‐generation optical systems, for example, silicon‐based on‐chip optical devices. Among the micro/nano‐optical devices, the optical switch becomes a key member, it allows or disallows the light propagation along a certain path by the “on” and “off” states. Thus, optical switches power a series of applications including on‐chip optical devices,^[^ ^2^ ^]^ optical logic circuits,^[^ ^3^, ^4^ ^]^ information storage,^[^ ^5^ ^]^ etc. Nowadays, optical switches in micro/nano‐scale have been widely realized by metasurface, waveguide, and optical films and attract great interest.^[^ ^2^, ^6^, ^7^ ^]^

Despite the rapid development in recent years, optical switches are still far from practical and large‐scale applications due to the lack of devices simultaneously with capacities of high contrast ratio, simple switch strategy, and low‐cost fabrication method. Many efforts have been made to overcome the challenges mentioned before. Traditional mechanical optical switches are first demonstrated by changing the optical path through the movement of optical fibers or optical components, thus achieving the purpose of on‐demand optical signal propagation, with the advantages of low insertion loss and high isolation. However, mechanical optical switches are bulky and hardly integrated, limiting their application in compact optical communications. To solve the drawbacks, electro‐optic and thermo‐optic effects have been introduced to achieve optical switches based on the refractive index change of materials under external excitation, overcoming the difficulties of large size and difficult integration,^[^ ^8^, ^9^, ^10^, ^11^ ^]^ but these switches require continuous external excitation to maintain the switching state and cause a high‐power consumption. In addition, the optical switch based on electro‐optic face problems of a low contrast ratio, but the thermos‐optic‐based one suffers the predicament of slow switching speed. To further optimize the performances of the device, some researchers have intensively investigated non‐volatile phase change materials (PCMs) to realize the optical switch simultaneously with the superior capacities of low power consumption, high contrast ratio, and rapid switching process.^[^ ^12^, ^13^, ^14^, ^15^, ^16^, ^17^ ^]^ In contrast to volatile vanadium dioxide, chalcogenic PCM does not require external excitation to maintain its phase change state. Ge_2_Sb_2_Te_5_ (GST), as a mature chalcogenic PCM, has been widely used to design optical switches by waveguides^[^ ^15^ ^]^ and metasurfaces.^[^ ^18^ ^]^ GST‐based optical switches are built up and show excellent switching speed and non‐volatile modulation thanks to the GST can be rapidly switched between the stable crystalline and amorphous state, and the reported switches are utilized in optical memory, optical communications, and neuromorphic computing during its phase change.^[^ ^19^, ^20^, ^21^ ^]^ However, the intrinsic loss of GST hinders the application of the related switches due to the limited contrast ratio. Recently, the Fabry–Perot (FP) cavity has also used in optical switches based on PCMs. Compared to the patterned optical switches, one based on the FP cavity, which consists of easily fabricated multilayer films, has achieved a high contrast ratio for its FP resonance. However, the contrast ratios of the reported optical switches are not fully optimized due to the optical loss of materials.^[^ ^22^, ^23^, ^24^ ^]^ Thus, designing optical switches with a high contrast ratio, fast switching, and low fabrication consumption is still challenging. Under the circumstances, a new type of PCM is needed to achieve high‐performance optical switches, where Ge_2_Se_2_Sb_4_Te (GSST) shows great advantages in optical contrast, response speed, low loss, and thermal stability,^[^ ^25^ ^]^ providing a fresh platform for achieving high‐performance optical switches and a new approach for the next generation of optical devices.

In this work, we develop and demonstrate a new optical switch using GSST‐based multilayer film, where the high contrast ratio and fast operating speed are obtained concurrently. Compared to the typical chalcogenic PCM GST, GSST has a smaller optical loss while retaining a larger optical contrast between the amorphous and crystalline states, enabling a high level of contrast ratio in the GSST‐based optical switch. At the same time, we construct a switch using the FP cavity of multilayer film, which is one of the simplest configurations of micro/nano‐optical devices. By incorporating GSST with low optical loss into the FP cavity, the reflectance of the optical switch experiences a dramatic change due to the phase of GSST changing from amorphous to crystalline states by the heating process. Consequently, the “on” and “off” states are obtained by the phase change of GSST. Especially, the thickness of the multilayer of the optical switch is optimized, where the contrast ratio is greatly improved and reaches 2410/735 by simulation/experiment. Besides, the GSST crystallinity can be continuously modulated by the external temperature, further enabling the linear modulation of the contrast ratio. The as‐demonstrated optical switches not only inspire a new possibility of designing high‐performance optical devices at the micro/nanoscale but also power a series of next‐generation active optical systems.

## 2 Results and Discussion

**Figure 1:**
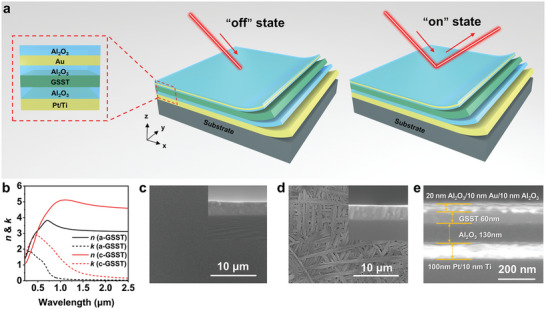
Device structure and SEM images. a) Schematic diagram of the GSST‐based optical switch. b) Refractive index and extinction coefficient of the amorphous GSST and the crystalline GSST. c,d) SEM images of GSST in the c) amorphous state and d) crystalline state. e) SEM image of the cross‐sectional profile of the GSST‐based optical switch.

**Figure** **1**a shows the schematic of the GSST‐based optical switch, which consists of seven layers deposited on a quartz substrate by physical vapor deposition. To realize the tunable interfering resonance, we designed the FP cavity and utilized GSST film to serve as one of the intermediate medium layers, where the PCM has a phase change temperature of ≈345 °C. In detail, a 100 nm Pt/10 nm Ti film is chosen as the bottom reflection mirror of the FP cavity. The upper intermediate medium layers consist of two dielectric layers, which from the top to bottom are GSST, and Al_2_O_3_, respectively. By tuning the thickness of GSST and Al_2_O_3_ layers, the FP resonance wavelength of the multilayer cavity device can be artificially adjusted, and the thickness of Al_2_O_3_ is identified as 130 nm. Another 10 nm Al_2_O_3_ layer is covered on the GSST layer to serve as a diffusion barrier. Moreover, a 10 nm thick semi‐reflection Au film acts as another mirror of the FP cavity to achieve multi‐beam interference. Finally, a 20 nm thick Al_2_O_3_ film is adopted both as the protective and anti‐reflective layer of the multilayer structure. As for the amorphous state, the GSST layer allows the near‐infrared (NIR) light to transmit freely through the intermediate medium layers due to its low extinction coefficient. The light is reflected between the bottom Pt/Ti mirror and the upper semi‐reflection Au layer, resulting in destructive interference and minimal reflected light. Thus, the multilayer cavity device is in the “off” state (Figure 1a). When changing into the crystalline state, the refractive index and extinction coefficient of GSST increase. The former would lead to a red‐shift of the resonance, while the latter would weaken the destructive interference. Thus, the multilayer cavity device transforms to the “on” state. Figure 1b depicts the refractive index and extinction coefficient of GSST under the amorphous and crystalline states, demonstrating an obvious refractive index difference in the NIR regime that leads to excellent performance of the optical switch. Figure 1c,d illustrate the scanning electron microscope (SEM) images of the amorphous and crystalline GSST films, in which the phase change process is accomplished by annealing at slightly above 345 °C for 10 min, we can observe a regular grain arrangement of crystalline GSST film compared to the uniform amorphous film. The sample diagram of the multilayer thin film structure is shown in Figure 1e, showing that each layer of the device has a smooth surface and a thickness consistent with the design.

**Figure 2:**
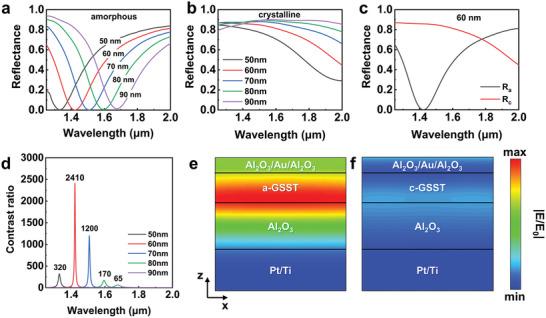
Simulated spectra and electric fields of the optical switches. a,b) Simulated reflectance spectra of the optical switches with different thicknesses of the GSST layer while GSST is in the (a) amorphous state and (b) crystalline state. c) Simulated reflectance spectra of the optical switch with a 60 nm‐thick GSST layer. d) Simulated contrast ratios of the optical switches with different thicknesses of the GSST layer. e,f) The simulated electric field of the optical switch with a 60 nm‐thick GSST layer in the (e) amorphous and (f) crystalline states.

We have utilized the principle of the FP cavity and the phase transition of GSST for the design of the device with a high contrast ratio. Numerical simulations were carried out using the finite difference time domain (FDTD) method to optimize the parameters of the multilayer thin films. **Figure** **2**a,b depicts the transmission of the multilayer with various thicknesses of amorphous and crystalline GSST, respectively. As for the amorphous state, the switches show a resonance in the NIR band, which is correspondingly represented by a very low reflectance (near zero) at a specific wavelength (corresponding to the optical switching “off” state), whereas the crystalline sample generally shows a high reflectance of more than 80% at the same wavelength (corresponding to the optical switching “on” state). The above‐mentioned effect indicates that the multilayer film can be used in high contrast ratio optical switches via the phase change of the GSST. The resonant wavelength is related to the thickness of the GSST film, the minimum reflectance can be regulated from 1330 to 1670 nm by easily increasing the film thickness, meaning that we can adjust the operating frequency of the switch.

To represent the performance of the optical switch, we introduce the contrast ratio for characterization. The contrast ratio is defined by *con = R_c_/R_a_*, where *R_c_* and *R_a_* are reflectances of the crystalline and amorphous state. As an example, for the sample with the GSST layer thickness of 60 nm in Figure 2c, when the sample is converted from the amorphous to the crystalline state, the simulated modulation depth is 85% and the contrast ratio reaches 2410 at 1420 nm. Figure 2d illustrates the contrast ratios for switches with GSST layers of distinct thickness, where the optical switch with a 60 nm‐thick GSST layer shows the highest contrast ratio. Notably, the contrast ratio is mostly beyond 100 and covers a wide band including several communication bands. The origin of the high contrast ratio can be uncovered by the field distribution of the multilayer, and the simulated electric fields along the x‐z plane corresponding to the 60 nm sample in amorphous and crystalline states are shown in Figure 2e,f, which show that it is in line with the designed results expected, and the light is localized within the FP cavity.

**Figure 3:**
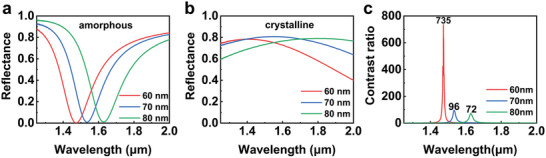
Experimental characterization of the optical switch. a,b) Measured reflectance spectra of the optical switches with different thicknesses of the GSST layer while GSST is in the (a) amorphous state and (b) crystalline state. c) Calculated contrast ratios of the optical switches with different thicknesses of the GSST layer.

To verify the performance of the designed GSST‐based optical switch, we prepared samples of GSST with thicknesses of 60, 70, and 80 nm, respectively. The reflectance spectra are collected both in their amorphous and crystalline states by a Fourier transform infrared spectroscopy (FTIR), and the spectra are shown in **Figure** **3**a,b. Similarly, to the simulation, a redshift of the reflected dip can be found in the amorphous state switches, but their intensity maintains a low level of nearly zero (Figure 3a). When it comes to the crystalline state, high reflectance is observed, indicating the intensity of the light propagation can be controlled over a large range. Figure 3c further shows the contrast ratio calculated for the optical switches with different GSST thicknesses. High contrast ratios of 735, 96, and 72 are obtained for the devices with GSST in thicknesses of 60, 70, and 80 nm. Especially, the high contrast ratio that appears in the switch of 60 nm‐thick GSST is far beyond the previous report.^[^ ^24^, ^26^, ^27^, ^28^, ^29^, ^30^ ^]^ Meanwhile, the GSST in our optical switches is a continuous film, meaning the switch can inherit the superior properties of response speed and thermal stability of the material. The operating bands of the switches range from 1460 to 1630 nm, which are the most important bands in optical communication. There is no doubt that the operating wavelength can be continuously tuned in this band by adjusting the thickness of GSST, and the high‐performance devices working in S, C, and L bands can be potentially designed with the FP optical switches. Although the experimental contrast ratio is smaller than the simulation, the evolution trend with GSST thickness is still the same, and other difference between the experiment and simulation comes from the imperfection of the sample fabrication and measurement.

When GSST is converted from the amorphous to the crystalline state, its dielectric constant undergoes a continuum change with the increase of crystallization degree, inspiring a possibility of achieving a tunable contrast ratio. The proposed switches with high contrast ratios are greatly suitable for the achieving of multi‐bit switches. To achieve the target, we have calculated the optical parameters of GSST in the partially crystallized state using the linear interpolation of the complex dielectric constant:^[^ ^31^ ^]^

(1) $$\varepsilon_{i}^{\prime }=\varepsilon_{a}^{\prime }+s\left({\varepsilon^{\prime }}_{c}-{\varepsilon^{\prime }}_{a}\right)$$

(2) $$\varepsilon_{i}^{\prime \prime }=\varepsilon_{a}^{\prime \prime }+s\left({\varepsilon^{\prime \prime }}_{c}-{\varepsilon^{\prime \prime }}_{a}\right)$$

where *s* takes values from 0 to 1, refers to the ratio of crystallized degree, and *ε’* and *ε”* are the real and imaginary parts of the dielectric constant, where the subscript *a, c*, and *i* represent the amorphous, crystalline, and intermediate states of GSST, respectively. The refractive index (*n*) and the extinction coefficient (*k*) can be further obtained with the following relationship:

(3)

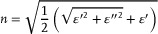



(4)

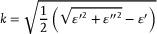




**Figure 4:**
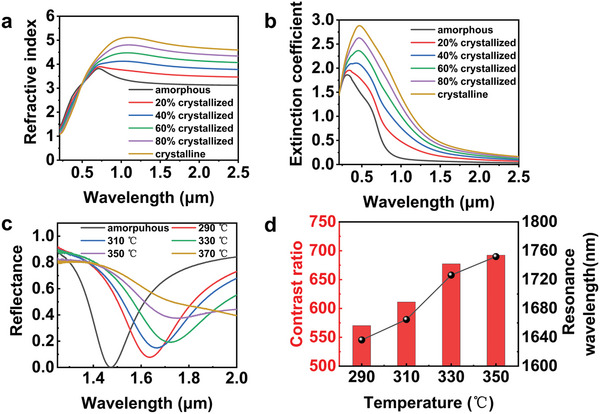
Thermal modulation of the GSST‐based optical switch. a) Refractive index and b) extinction coefficient of the GSST alloy for different crystallization states. c) Measured reflectance spectra of the GSST‐based optical switch annealed at different temperatures. d) Calculated contrast ratio and measured resonance wavelength associated with gradient temperatures.

The results are shown in **Figure** **4**a,b, which present curves of the refractive index (n) and extinction coefficient (k) for GSST across different crystallization states. It is clear that the optical parameters are undergoing a continuum change with the crystallization process, which lays the foundation for the thermal modulation of the device.

Experimentally, we can prepare GSST with different crystallization degrees by simply introducing a thermal process, where GSST has a low phase change temperature of 345 °C and the ability to accurately regulate to the intermediate state. By placing the samples in a rapid annealing furnace and setting them to anneal at different temperatures for 3 min, the GSST was partially crystallized to complete the thermotropic phase transition modulation of the samples.^[^ ^32^ ^]^ When the temperature is below 290 °C, the GSST does not undergo a phase change process and the optical switch is maintained in the “off” state, thus we concentrate on the devices that are thermally processed by the temperature above 290 °C. The reflected spectra of the partially crystallized switch are collected and shown in Figure 4c, with the temperature increases, the spectral dips of the reflectance spectra are gradually red‐shifted, indicating the resonant wavelength increases, and the reflectance at the response wavelength gradually increases, which indicates that our device is capable of thermotropic phase transition modulation. In addition, the curve corresponding to 370 °C fits well with the spectrum of the crystalline sample in Figure 3b, indicating that the sample annealed at 370 °C underwent complete crystallization, suggesting that the sample can complete the full‐segment thermal modulation from amorphous to crystalline state. Figure 4d shows the variation of the response wavelength and contrast ratio with temperature for the 60 nm sample, and the response wavelength grows roughly linearly with temperature, indicating that the device can complete an accurate quantitative thermal modulation. Meanwhile, the contrast ratio of the optical switch also increases with the annealing temperature, demonstrating that our optical switch is capable of accomplishing active thermal modulation.

**Table** **1** summarizes the comparison of as‐fabricated optical switches with the previous reports. Compared to the previous switches, our work introduced the low‐loss GSST as the active material, which brings many advantages in configuration and contrast ratio. As for the configuration, the switch is designed as a multilayered FP cavity, which can dramatically shrink the fabrication cost than those switches consisting of metasurface or metamaterials. Besides, the modulation approaches, working band, and propagation path maintain the mainstream model of the previous reports for the co‐polarized switches that regulate the reflected NIR beam are most popular in optical devices. Most importantly, the contrast ratio of 735 highlights the core advantage of our switch, which is far beyond the other devices that are usually smaller than 10. Moreover, in comparison with other multilayered optical switches, our optical switch breaks the constraints on materials and realizes superior contrast ratio performance. The properties of high performance and low fabricating cost enable one to design optical devices in micro/nanoscale with advanced functions and pave the way for the practical applications of optical switches. In the next work, we hope to introduce diversified modulation means, such as laser or electrical modulation to further enhance the application scenarios of optical switches.

**Table 1:** Comparison of the optical switch proposed in this work with previous literature.

Materials	Structure	Modulation approaches	Working band	Propagation path	Contrast ratio	Reference
GST‐225	Metamaterial	Co‐polarization	NIR	Transmittance & Reflectance	≈4	Ref. [26]
GST‐326	Metasurface	Co‐polarization	Visible	Reflectance	≈4.5	Ref. [27]
ITO	Metasurface	Cross‐polarization	NIR	Reflectance	≈4	Ref. [28]
GST‐225	Metasurface	Co‐polarization	NIR	Reflectance	≈10	Ref. [29]
GST‐225	Metasurface	Cross‐polarization	NIR	Transmittance	≈7.5	Ref. [30]
GeTe& GST‐225	Multilayer films	Co‐polarization	Visible	Reflectance	≈30	Ref. [24]
GSST	Multilayer films	Co‐polarization	NIR	Reflectance	≈735	This work

## 3 Conclusion

In this work, we designed and experimentally verified a GSST‐based near‐infrared optical switch with a high contrast ratio. The device is designed based on the principle of FP resonance, and GSST, a PCM with high optical contrast and low loss, is introduced to enhance the performance of the optical switch. Numerical simulations show that the optical switch is worked by the phase change induced localized electric field change, and the contrast ratio reaches 2410 under the GSST thickness of 60 nm. Both the operating wavelength and contrast ratio can be modulated by the material's thickness. Experimentally, we prepared a series of optical switches with different GSST thicknesses and characterized their performance. All the results show high contrast ratios over 1460–1630 nm wavelength, which involves the most important communication frequency including “S,” “C,” and “L” bands. Where the switch with a 60 nm‐thick GSST layer has the highest contrast ratio, reaching an extreme value of 735. In addition, the multilevel nonlinear modulation of the optical switches can be realized by using thermal processing to modulate the crystallinity of GSST.

In conclusion, our optical switches not only combine the advantages of simple configuration and low fabrication cost but also exhibit excellent contrast ratio performance. We believe that our optical switches have great potential for applications in areas such as optical data storage, information encryption, or dynamic optical modulation, and inspire the development of a range of advanced reconfigurable optical components on the micron/nanoscale. Subsequent structural optimization and introduction of electrical modulation to the GSST‐based optical switches to obtain faster switching speeds will further broaden the applications of optical switches in optical real‐time computing and optical communications. Furthermore, changing the material can further modulate the operating wavelength and optimize the performance of the optical switch, for example, 1310 nm, the fundamental band for communication, thus broadening the practical applications of optical switches.

## 4 Experimental Section

### Numerical Simulation

The optical reflectance spectra of the designed optical switch were simulated by the FDTD method. The periodic boundary condition of the model unit cell was adopted along with both the *x‐* and *y‐*direction, while perfectly matched layers (PML) were set along the *z*‐direction. Plane waves were launched incident along the *z*‐direction, and the wavelength was set from 1.25 to 2 µm. A monitor along the *x‐y* plane was set to record the needed data of reflectance spectra, while a monitor along the *x‐z* plane to acquire data of the electrical field. During the simulation, the optical parameters of GSST were acquired by an ellipsometer, and those of other materials were obtained from the Handbook of Optical Constants of Solids I – III by E. Palik and the CRC Handbook of Chemistry & Physics.

### Sample Fabrication

The optical switch device was implemented through a series of standard fabrication processes. Before fabrication, a 2 cm × 2 cm quartz substrate underwent ultrasonic cleaning in acetone, isopropanol, and ultrapure water, which was then dried using nitrogen flow. The devices consisting of multilayer thin film were subsequently fabricated by electron beam evaporation (EBE) and magnetron sputtering (MS). Among them, the Pt/Ti, Au, and Al_2_O_3_ layers were deposited by EBE, and the GSST layer was deposited by MS. The deposition of GSST was performed using a synthesized single target at room temperature with argon gas flow rate at 15 sccm, growth pressure at 0.007 mTorr, and sputtering radio frequency power of 70 W. All films used in the optical switches have a thickness error of ≤ 3%. The heating process of the optical switches and GSST films is accomplished in an annealing furnace under an argon gas atmosphere.

### Measurement Setup

The refractive index and extinction coefficient of GSST were measured on spectroscopic ellipsometry (RC 2, Woollam) with the collected band set from 0.21 to 2.5 µm. The SEM photographs were taken on Hitachi Regulus 8230 with an accelerating voltage of 7 kV. The sample scan time and background scan time were set to 128 scans. The experimental reflectance spectra were measured on an FTIR (Vertex 80, Bruker), whose resolution was set as 2 cm^−1^ and the test band of the spectra was set from 1.25 to 2 µm.

## Conflict of Interest

The authors declare no conflict of interest.

## Data Availability

The data that support the findings of this study are available from the corresponding author upon reasonable request.
